# C-856-04. Implementation of the Multi-tier Approach to Psychological Intervention After Traumatic Injury (MAPIT) Program in Burns

**DOI:** 10.1093/jbcr/irag033.096

**Published:** 2026-04-06

**Authors:** Sarah A Stoycos, Laura Ramos, Haig A Yenikomshian, Spencer Liebman, Maxwell B Johnson, T Justin Gillenwater

**Affiliations:** Keck School of Medicine, University of Southern California, Los Angeles, CA; Los Angeles General Medical Center, Los Angeles, CA; Division of Plastic and Reconstructive Surgery, Keck School of Medicine, University of Southern California, Los Angeles, CA; Los Angeles General Medical Center, Los Angeles, CA; Division of Plastic and Reconstructive Surgery, Keck School of Medicine, University of Southern California, Los Angeles, CA; Division of Plastic and Reconstructive Surgery, Keck School of Medicine, University of Southern California, Los Angeles, CA

## Abstract

**Introduction:**

In 2013, a summary of the 2012 ABA burn quality consensus conference was published. In it, experts recommended universal PTSD and depression (Dep) screening in burn care given their prevalence and negative, long-term impact on outcomes. Twelve years later, significant advancements have been made, including expansion of ABA verification criteria related to mental health screening and referral (2019) and BCQP data fields for PTSD, acute stress, and Dep screening (2024). However, there is a gap in published implementation models or clinical practice guidelines to support burn centers in meeting these requirements. The current study evaluated the adaptation and implementation of tiers 1-2 (see Fig. 1) of the Multi-tier Approach to Psychological Intervention After Traumatic Injury (MAPIT) Program in a psychology-naive burn center in a large metropolitan area safety net hospital.

**Methods:**

Burn surgeons worked with hospital administration and burn psychology experts to create a FTE for a health psychologist with clinical and research expertise in traumatic injury and burns and program development and implementation. A collective goal was set to create a comprehensive psychological screen and treat approach for inpatient burns that satisfies ABA requirements and is scalable to other units and outpatient settings. Expert consensus based on current evidence base was used to select the MAPIT Program framework and to screen adult inpatient burn admissions for substance abuse (CAGE-AID: 4 yes/no items; urine and serum toxicology, U/S Tox), and post-injury risk for PTSD and Dep (Injured Trauma Survivor Screen: 9 yes/no items). Hospital and burn unit stakeholders were engaged to create the clinical pathway. Acceptability, feasibility, and applicability of the MAPIT Program were assessed.

**Results:**

Social work administered screens to patients during routine psychosocial assessment. 197 patients were screened; 97% (n = 191) completed the screens. 33.5% (n = 65) scored high-risk for post-injury PTSD, 30.2% (n = 59) scored high-risk for post-injury Dep, and 18% (n = 35) met high-risk for both. 28.4% (n = 55) screened positive for potentially problematic pre-burn substance use on the CAGE-AID. U/S Tox levels were captured in the dataset for 131 patients, 51.9% (n = 68) resulted positive (excluding likely iatrogenic positives) and 44.1% (n = 30) of those also self-reported substance abuse on the CAGE-AID.

**Conclusions:**

An interdisciplinary team-based approach that leverages hospital stakeholders and existing resources facilitated implementation of MAPIT. Routine, universal mental health screening of admitted burn patients is feasible, acceptable, and applicable to acute burn care.

**Applicability of Research to Practice:**

A significant minority of burn patients are at high risk for post-injury PTSD, Dep, and substance abuse. Early identification of patients at high risk is essential to guide targeted intervention and optimization of limited mental health resources.

**Funding for the study:**

This work was supported by the National Institute of Mental Health (1K23MH141296-01). The contents do not necessarily represent the policy of NIMH and endorsement by the Federal Government should not be assumed.

